# Spectrum and resistance in bacterial infections of the ocular surface in a German tertiary referral center 2009–2019

**DOI:** 10.1007/s00417-022-05721-7

**Published:** 2022-06-10

**Authors:** Mathias Roth, Paul Goerke, Christoph Holtmann, Andreas Frings, Colin R. MacKenzie, Gerd Geerling

**Affiliations:** 1 Department of Ophthalmology, Heinrich-Heine University Duesseldorf, Moorenstr. 5, 40225 Duesseldorf, Germany; 2 Institute of Medical Microbiology and Hospital Hygiene of the University Hospital Duesseldorf, Duesseldorf, Germany

**Keywords:** Ocular surface, Conjunctivitis, Keratitis, Bacterial spectrum, Antibiotic resistances, Contact lens

## Abstract

**Purpose:**

Aim of this study was to evaluate the frequencies, trends, and antibiotic resistance of bacteria collected from ocular surface or contact lens material in a German tertiary referral center from 2009 to 2019.

**Methods:**

Microbiological data from 2009 to 2019 was analyzed. Culture-dependent microbial identification and analysis of antibiotic sensitivity was completed by the Institute of Microbiology. Statistical analysis of age- and sex-specific differences as well as changes in the microbial spectrum and resistance over the study period was performed with GraphPad Prism 9.0 applying nonparametric tests (level of significance: *p* ≦ 0.05).

**Results:**

A total of 6361 specimens were analyzed. Positivity rate was 18.6%. Sixty-three percent (*n* = 680) of the bacterial isolates were derived from ocular surface and 37% (*n* = 399) from contact lens material. The ratio of gram-negative bacteria was significantly higher in contact lens material. Multiresistant bacteria showed a significant increase with patient age (*p* < 0.0001). An overall increase in resistance to levofloxacin (*p* = 0.0239) was detected. Only 2.4% and 3.1% isolates were resistant to a combination of moxifloxacin and gentamicin, respectively, levofloxacin and gentamicin.

**Conclusions:**

The reported bacterial spectrum is similar to comparable centers. Our data show that it should not be assumed that the newest classes of antibiotics have the best efficacy or lowest resistance levels. In suspected bacterial conjunctivitis, we propose using gentamicin as first-line therapy. In therapy refractive cases and in involvement of the cornea, we recommend a combination of gentamicin and ofloxacin or moxifloxacin. Overall, the evaluated organisms showed good sensitivity to the regularly used antibiotics.

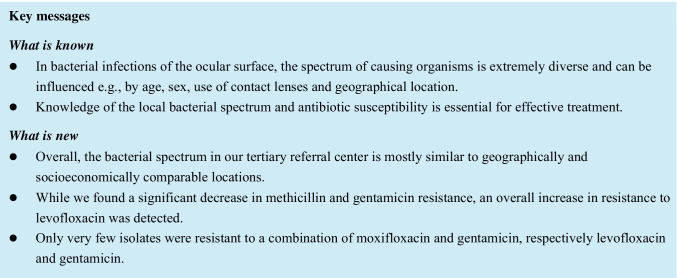

## Introduction

Microbial keratitis can result in severe loss of visual acuity and represents one of the most common causes of corneal blindness [1, 2]. In presumed bacterial infections of the ocular surface, empiric antibiotic treatment is commonly initiated without identification of the causative organism. The bacterial spectrum in ocular surface infections can be extremely diverse [3]. This spectrum can be influenced by several parameters, such as age, sex, use of contact lenses, geographical location, and even the season of the year [4–8]. Widespread use of systemic broad-spectrum antibiotics can lead to a change in the bacterial spectrum and an increase in antibiotic resistance. Knowledge of the current local bacterial spectrum and antibiotic susceptibility is essential for effective treatment in ocular infections. Therefore, the aim of this study was to determine and evaluate the frequencies, trends, and antibiotic resistance of bacteria collected from ocular surface or contact lens material in a German tertiary referral center from 2009 to 2019. Furthermore, we analyzed the correlation of bacteria and drug resistance with the age and sex of the patients.

## Material and methods

Before initiation of the study, approval was obtained from the Ethics Committee of the Medical Faculty of Duesseldorf (file number 4797). The study adhered to the tenets of the Helsinki Declaration.

All microbiological cultures taken from the ocular surface or contact lens material at the Department of Ophthalmology of the University of Duesseldorf — a tertiary referral center for corneal disease — between 2009 and 2019 were identified via the database of the Institute of Microbiology, University of Duesseldorf. Intraocular isolates were excluded. As the origin of the swabs (conjunctiva vs. cornea; contact lens vs. contact lens container) could not always be identified with certainty, the respective specimens were grouped as *ocular surface* (conjunctiva and cornea) and *contact lens material* (contact lenses, contact lens container, and contact lens solution).

Standard microbiological culture methods and media for bacteria and fungi were used for the culture of specimens. The spectrum of microbes from positive cultures and their sensitivity and resistance to antibiotics were evaluated. Depending on the type of isolated organism, antibiotic susceptibility to oxacillin, gentamicin, levofloxacin, ciprofloxacin, moxifloxacin, cotrimoxazole, erythromycin, clindamycin, vancomycin, and fusidic acid was tested. Antimicrobial susceptibility testing was performed using the semiautomated Vitek 2 system (bioMérieux, Nürtingen, Germany) and, when necessary, with gradient diffusion strip tests (Liofilchem, Abruzzo, Italy). Breakpoints were defined by the current EUCAST table at the time of interpretation. As tissue concentrations of antibiotic eyedrops in the ocular surface are considered to be high, “*I*” or “susceptibility with increased exposure” was counted as “susceptible,” according to current EUCAST recommendations [9]. The number of concurrent resistances per organism was evaluated for single substances as well as antibiotic classes: beta-lactams (oxacillin), aminoglycosides (gentamicin), fluoroquinolones (levofloxacin, ciprofloxacin, and moxifloxacin), cotrimoxazole, macrolides (erythromycin), lincosamines (clindamycin), glycopeptides (vancomycin), and fusidic acid. The age and sex of the respective patients, as well as the year of sample collection, were evaluated regarding possible correlations with a change in the spectrum and resistances.

### Statistical evaluation

Statistical analysis was performed using Prism 9.0.0 (GraphPad, La Jolla, CA, USA). Data are presented descriptively as the mean ± standard deviation. Groups were analyzed with ANOVA or the Mann–Whitney test. The chi-test and Spearman’s *R* were used to investigate correlations. As a measure of trending, the Cochrane-Armitage test was used. A simple linear regression was used to plot the trend lines. The *p* values ≤ 0.05 were considered statistically significant.

## Results

From 2009 to 2019, a total of 6361 specimens were analyzed. Ninety percent (*n* = 5724) of the specimens were derived from the ocular surface, and 10% (*n* = 637) were derived from contact lens material. In total, 1181 specimens (18.6%) had a positive culture result, of which 1079 (91.4%) were bacterial and 102 (8.6%) were fungal isolates. Of the positive bacterial isolates, 63% (*n* = 680) were derived from ocular surface samples, and 37% (*n* = 399) were derived from contact lens material.

The annual positive isolation rate ranged from 12.7% (2018) to 28.4% (2010), with a decreasing trend (*p* = 0.0058). Candida was the main fungus detected (55%), followed by Fusarium (31%) and others (14%). For further analysis, fungal isolates were excluded, and only the 1079 specimens with positive bacterial results were analyzed. The average age of patients with a bacterial isolate was 46 ± 23 years (median (IQR 25%, IQR75%): 44 (27; 65) years). Fifty-two percent of the patients were female (*n* = 562), and 48% were male (*n* = 517).

In total, 45.5% (*n* = 491) of the isolates were gram positive. Most bacteria belonged to the order Enterobacterales (19.4%, *n* = 209), followed by Coagulase-negative staphylococci (CoNS) in 14.1% (*n* = 152), *Pseudomonas* species in 18.1% (*n* = 196), *Staphylococcus aureus* in 16.0% (*n* = 173), streptococci in 4.6% (*n* = 50), and *Haemophilus influenzae* in 4.4% (*n* = 47). The remaining 23.4% (*n* = 253) were diverse bacterial species. Table 1 shows the distribution of the isolated species. Analyzed by the species subgroups, there were significant differences depending on sex (Enterobacterales, *Pseudomonas* species, and *Staphylococcus aureus*) and age (Enterobaterales, *Pseudomonas* species, streptococci, and *Staphylococcus aureus*) (see Table 1). Regarding Gram staining, there was no sex-dependent difference, whereas patients with gram-positive isolates were significantly older (52 years ± 24) than patients with gram-negative isolates (41 years ± 20; *p* < 0.001).

**Table 1:** Overview of the bacterial spectrum and resistance

	Overall	CoNS	*Pseudomonas* spp.	*Enterobacterales*	*H. influenza*	*Streptococci*	*S. aureus*	Other	Gram +	Gram −
Overall, *n*/%	**1079/100%**	152/14%	196/18%	209/19%	47/4%	50/5%	173/16%	252/23%	491/46%	588/54%
Trend over time, *p*	**–**	ns	0.0039	ns	0.003	ns	ns	ns	ns	ns
Sex (female), *p*	**48%, –**	53%, ns	59%, < 0.001	58%, < 0.001	47%, ns	46%, ns	45%, < 0.001	48%, ns	49%, ns	55%, ns
Age (mean in years ± SD), *p*	**46 ± 23, –**	46 ± 22, ns	40 ± 20, < 0.001	39 ± 18, < 0.001	45 ± 25, ns	56 ± 23, < 0.001	56 ± 24, < 0.001	48 ± 24, ns	52 ± 24, < 0.001	41 ± 20, < 0.001
Sum of resistances (mean ± SD), *p*	**0,45 ± 1.12, –**	0.79 ± 1.71, < 0.001	0.06 ± 0.28, < 0.001	0.1 ± 0.37, < 0.001	0.21 ± 0.55, ns	0.2 ± 0.53, ns	1.12 ± 1.18, < 0.001	0.46 ± 1.34, ns	0.83 ± 1.5, < 0.001	0.13 ± 0.44, < 0.001
Origin of isolate										
Isolates ocular surface, *n* / %	**680/63%**	128/19%	77/11%	52/8%	47/7%	50/7%	169/25%	157/23%	440/65%	240/35%
Isolates lens material, *n*/%	**399/37%**	24/6%	119/30%	157/39%	**–**	**–**	4/1%	95/24%	51/13%	348/87%
Resistances										
Oxacillin resistance, *n*/%	**28/13%**	13/23%	–/–	–/–	–/–	–/–	0/0%	15/94%	28/13%	–/–
Gentamicin resistance, *n*/%	**35/7%**	12/21%	3/3%	0/0%	–/–	–/–	6/4%	14/18%	22/10%	13/4%
Levofloxacin resistance, *n*/%	**35/8%**	6/11%	0/0%	4/10%	1/2%	1/4%	8/5%	15/24%	29/12%	6/3%
Ciprofloxaxin resistance, *n*/%	**12/3%**	1/50%	1/1%	5/4%	1/2%	–/–	0/0%	4/5%	1/14%	11/3%
Moxifloxacin resistance, *n*/%	**42/9%**	6/11%	2/40%	7/7%	1/2%	0/0%	8/5%	18/23%	26/11%	16/8%
Cotrimoxazol resistance, *n*/%	**34/7%**	8/14%	6/100%	4/3%	6/16%	0/0%	2/1%	8/8%	11/5%	23/9%
Erythromycin resistance rate, *n*/%	**73/27%**	28/49%	–/–	-/–	1/25%	4/17%	28/19%	12/27%	72/29%	1/4%
Clindamycin resistance rate, *n*/%	**51/20%**	9/16%	–/–	–/–	–/–	5/19%	27/18%	10/38%	51/20%	0/0%
Vancomycin resistance rate, *n*/%	0/0%	0/0%	–/–	–/–	–/–	0/0%	0/0%	0/0%	0/0%	0/0%
Fusidic acid resistance rate, *n*/%	**25/12%**	21/40%	–/–	–/–	–/–	–/–	3/2%	1/6%	25/12%	–/–

Table 1 shows the resistance profiles for the respective species. The percentage refers to the total amount of isolates tested for the respective resistance. *ns* not significant

Patients with isolates from ocular surface samples were significantly older (53 years ± 24; range 0–99 years) than those with isolates from contact lens material (34 years ± 14; range 14–84 years). Additionally, the spectrum of isolated organisms differed significantly between those two groups. Sixty-five percent (*n* = 440) of the isolated organisms from the ocular surface were gram positive, while in lens-derived samples, 87% (*n* = 348) were gram negative. *Staphylococcus aureus* was almost exclusively isolated from the ocular surface. From contact lens material, Enterobacterales were isolated significantly more often, while Streptococci and *Haemophilus influenzae* were not found at all (Table 1).

**Fig. 1:**
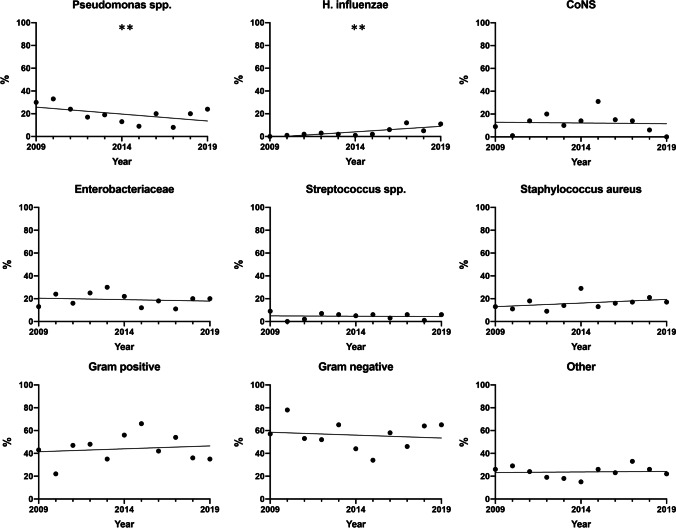
The Cochrane-Armitage trend test revealed a significant decrease in *Pseudomonas* over time (*p* = 0.0039) and an increase in *Haemophilus influenzae* (*p* = 0.0030)

In the evaluation of the separate species, the test for trend for the separate species showed a decrease in Pseudomonas (*p* = 0.0039) and an increase in *Haemophilus influenzae* (*p* = 0.0030) over time. The other species, including overall gram-positive and gram-negative isolates, did not change significantly over time (Fig. 1).

**Fig. 2:**
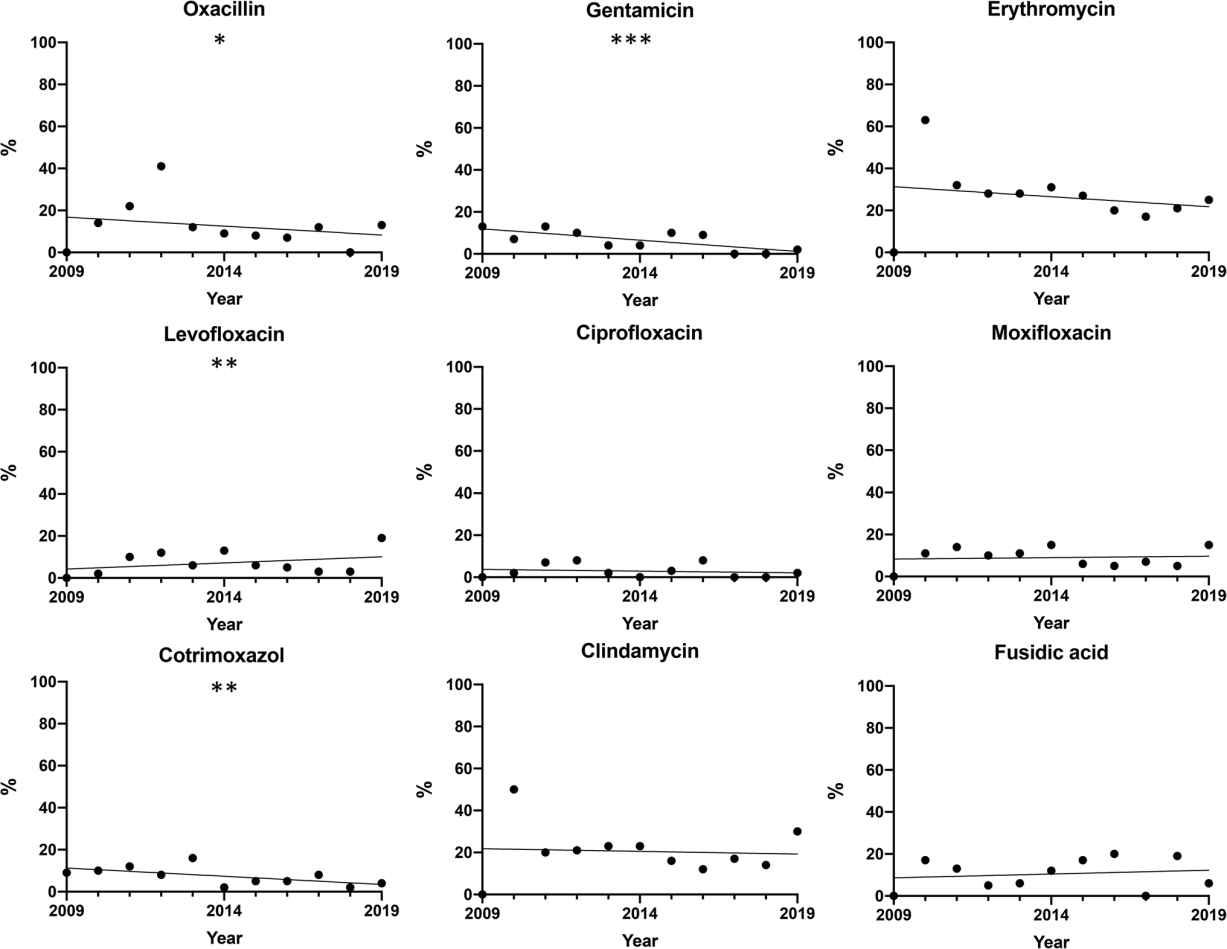
In the analysis of the resistances, the test for trend showed a significant decrease in methicillin resistance (*p* = 0.0119), gentamicin resistance (*p* < 0.0001), and cotrimoxazol resistance (*p* = 0.0024). The levofloxacin resistance increased (*p* = 0.0239)

Of the 1079 bacterial isolates, 245 isolates (22.7%) were resistant to at least one of the tested antibiotics. In each of these isolates, resistance to a mean of 1.97 ± 1.59 antibiotics and 1.71 ± 1.16 different antibiotic classes was found. The maximum number of antibiotics to which a single organism was resistant was 9 single substances and 6 antibiotic classes. There was not a single vancomycin-resistant isolate. The number of multiresistant bacteria (single substances, as well as antibiotic classes) showed a significant increase with patient age (*p* < 0.0001, single substance: *r* = 0.14; antibiotic classes: *r* = 0.12). Neither a sex-dependent difference nor a change over time throughout the observation period was found regarding the number of multiresistant organisms for single substances, as well as antibiotic classes. Interestingly, over time, a significant decrease in resistance was found for oxacillin/methicillin (*p* = 0.0119), gentamicin (*p* < 0.0001), and cotrimoxazol (*p* = 0.0024), while resistance to levofloxacin increased (*p* = 0.0239) (Fig. 2).

In comparison to all other organisms, *Staphylococcus aureus* showed the highest number of concurrent resistances to single substances as well as antibiotic classes, followed by Pseudomonas (see Table 1). Whereas all *Staphylococcus aureus* isolates were susceptible to oxacillin, 23% of the CoNS showed resistance to this antibiotic. Only 2.5% (*n* = 9) of the isolates tested for susceptibility of fluoroquinolones and aminoglycosides were resistant to a combination of levofloxacin and gentamicin, respectively, 3.1% (*n* = 11) to a combination of moxifloxacin and gentamicin. Around 60% of these isolates were CoNS (6/9, respectively, 6/11). While there were no sex-dependent differences in the resistance profiles, there was a correlation with patient age. On the one hand, patients with isolates resistant to erythromycin (resistant: 47 ± 24 years vs. susceptible: 57 ± 24 years; *p* = 0.002) and fusidic acid (resistant: 43 ± 26 years vs. susceptible: 55 ± 25 years; *p* = 0.024) were significantly younger than patients with susceptible isolates. On the other hand, patients with isolates resistant to oxacillin (resistant: 63 ± 26 years vs. susceptible: 46 ± 22 years; *p* = 0.037) and levofloxacin (resistant: 63 ± 25 years vs. susceptible: 48 ± 24 years; *p* < 0.001) were significantly older than patients with isolates susceptible to those antibiotics.

## Discussion

### Isolation rate

Our overall yield of positive cultures of 18.6% is comparable to the study by Silvester et al. In their study, 15.8% of approximately 8200 conjunctival swabs were considered positive [10]. In contrast, most other studies including only material from corneal scrapings show higher rates of positive cultures, ranging from 32.6 to 61.0% [6, 11–15]. This difference could be explained by the higher bacterial load in corneal samples, which are arguably routinely taken only in later stages of a microbial infection when a corneal infiltrate is clinically apparent. Conjunctival swabs are not only taken from a tissue with usually no infiltrate but may in some cases have been performed in the absence of any corneal infiltrate, e.g., due to increased tearing. Additionally, some of our patients with conjunctivitis may have been already treated with a topical antibiotic prior to sampling. In contrast, a specimen for culture is more likely taken before therapy in patients with keratitis. Tan et al. analyzed samples of microbial keratitis and found a decline in positive cultures over the study period, as we did in our study [6]. They speculated that this might be a consequence of increased antibiotic usage before sampling or an increase in the number of unnecessary samples in noninfective cases [6].

### Spectrum of pathogens

In spite of difficulties comparing the bacterial spectrum because of the varying inclusion criteria [6, 10–14, 16, 17], geographic variations in the bacterial spectrum in ocular surface-based isolates have been described [5]. The ratio of gram-positive and gram-negative bacteria in our study was similar to geographically and socioeconomically comparable locations, such as Basel (Switzerland), Manchester (UK), New York (USA), and St. Louis (USA) [6, 18–20]. In line with our results, Steger et al. in Basel found a significantly higher ratio of gram-negative bacterial isolates in contact lens samples (71%) than in of conjunctival swabs of patients with bacterial keratitis (37%) [16]. This association between gram-negative bacterial infection and wearing contact lenses is well known, and possibly caused by biofilm formation [21–24].

While we were not able to find any other data regarding an increase in *Haemophilus influenzae* as in our analysis, in line with our results, Soleimani et al. in Tehran (Iran) reported a significant decrease in Pseudomonas in patients with infectious keratitis in a 6-year period at a referral center in Tehran (Iran) [14]. In contrast, Hsu et al. found a significant increase in *Pseudomonas aeruginosa* in an analysis over 15 years in St. Louis (USA) [19]. While in our analysis, there was no change over time in the ratio of gram-positive to gram-negative bacteria, there are controversial results reported in other studies [11, 14, 21].

### Antibiotic resistance

Our data show an overall resistance against levofloxacin of 8%, ciprofloxacin of 3%, moxifloxacin of 9%, and gentamicin of 7%. These results regarding fluoroquinolones and aminoglycosides, the most frequently used antibiotics, are comparable with those of other studies [12, 21, 25]. But, while Petrillo et al. described gentamicin, ciprofloxacin, and moxifloxacin resistance rates in CoNS of 72.8%, 84.2%, and 84.2%, respectively [26], they were lower in the present study (gentamicin 21%, ciprofloxacin 50%, and moxifloxacin 9%). Asbell et al. found 35% of the *S. aureus* isolates and 49% of the CoNS isolates to be methicillin resistant [27], and Petrillo et al. reported 23.7% of *S. aureus* and 61.7% CoNS isolates to be methicillin resistant [26]. We also observed a relatively high rate of methicillin resistance in CoNS (23%), but — at a current local MRSA rate of 5% — did not see any resistance in *S. aureus*, which is comparable to the results of Lichtinger et al. [21]. While we found a significant decrease in methicillin resistance, the reported results in the literature regarding this last-resort antibiotic are controversial [11, 26–28]. Also, our trend toward a low rate of resistance to gentamicin is in contrast to several comparable studies [11, 13, 14, 21, 26].

Special consideration should be taken in the choice of fluoroquinolone, as this study demonstrates a substantial difference in the susceptibility between ciprofloxacin (2nd-generation fluoroquinolone) versus 3rd- (levofloxacin) and 4th-generation (moxifloxacin) fluoroquinolone. In agreement with many other studies, we found an increasing resistance to fluroquinolones [14, 19, 29–33]. This trend can probably be explained by the frequent use of fluroquinolones as first-line therapy in bacterial conjunctivitis and keratitis. A rising resistance over time, especially for 4th-generation fluoroquinolones, as reported by Chang et al., must be acknowledged and observed with caution [33]. New fluoroquinolones, such as besifloxacin, a 4th-generation fluoroquinolone developed specifically for topical ocular indication (FDA approval 2009), might be a valuable alternative but is not yet available in our country [34]. In an Australian study by Watson et al., the resistance to chloramphenicol in combination with ciprofloxacin/ofloxacin was only 1.3% and thus might also be considered as an alternative empirical treatment [35]. Although the risk of chloramphenicol-induced agranulocytosis with less than one per million treatment courses is very low, it might be the reason why it is not regularly used in Germany [36, 37], while it is a widely used therapy in other countries.

### Influence of age and gender

In our study, as well as in the comparable literature, females and males were relatively equally distributed in the overall cohort [12, 15, 21, 22, 26–28, 38]. However, while we found sex-specific differences for Enterobacterales, Pseudomonas, and *Staphylococcus aureus*, in the other studies, no such difference was identified [12, 15, 21, 22, 26–28, 38].

Several studies report correlations between age and the spectrum of isolates as well as antibiotic resistance, albeit with large variation. We suggest that this may be correlated with the prevalence of contact lens wear in the different age groups, as described by Bograd et al. [20]. Interestingly, we did not find a correlation between young age and *Haemophilus* spp., as might have been expected according to the literature [27, 39]. In addition, we confirmed the results of other studies that resistance correlates with age, e.g., the higher incidence of methicillin resistance in *S. aureus* and CoNS in older patients [27]. The need for nursing home and hospital care has been suspected to be a risk factor for this [17].

### Limitations

A limiting factor of our and other studies may be the relatively low positivity rate and the limited number of resistances for the studied substances. Also, the intracorneal concentrations of topically and at high frequency applied antibiotics are known to be very high. They are far higher than those achieved by systemic drug administration, on which minimal inhibition concentrations for susceptibility evaluation are based in vitro [5]. Thus, the actual ophthalmological clinical susceptibility rate is likely to be higher than the rate reported in vitro [5, 20].

## Conclusions

The reported bacterial spectrum is similar to comparable centers. The most common bacterial pathogen were Enterobacterales, followed by CoNS and Pseudomonas species, which were significantly more often isolated from contact lens material than from the ocular surface. *Staphylococcus aureus* showed the highest degree of multiple antibiotic resistance, followed by Pseudomonas. Our data show that it should not be assumed that the newest classes of antibiotics always have the best efficacy or lowest resistance levels. In suspected bacterial conjunctivitis, we propose to use gentamicin as first-line therapy. In therapy refractive cases and in involvement of the cornea, we recommend the use of a combination of gentamicin and ofloxacin or moxifloxacin. If there is clinical suspicion for methicillin resistance, additional use of vancomycin is recommended. Overall, the evaluated organisms showed good sensitivity to the regularly used antibiotics.

## References

[1] Roth M, Daas L, Renner-Wilde A et al (2019) The German keratomycosis registry: initial results of a multicenter survey. Ophthalmologe 116. 10.1007/s00347-019-0871-9 10.1007/s00347-019-0871-9 30810837

[2] Roth M, Holtmann C, Daas L (2021). Results from the German Fungal Keratitis Registry: significant differences between cases with and without a history of contact lens use. Cornea.

[3] Bartimote C, Foster J, Watson S (2019). The spectrum of microbial keratitis: an updated review. Open Ophthalmol J.

[4] Gentile RC, Shukla S, Shah M (2014). Microbiological spectrum and antibiotic sensitivity in endophthalmitis: a 25-year review. Ophthalmology.

[5] Grzybowski A, Brona P, Kim SJ (2017). Microbial flora and resistance in ophthalmology: a review. Graefe’s Arch Clin Exp Ophthalmol.

[6] Tan SZ, Walkden A, Au L (2017). Twelve-year analysis of microbial keratitis trends at a UK tertiary hospital. Eye.

[7] Teweldemedhin M, Gebreyesus H, Atsbaha AH (2017). Bacterial profile of ocular infections: a systematic review. BMC Ophthalmol.

[8] Ting DSJ, Ho CS, Cairns J (2021). Seasonal patterns of incidence, demographic factors and microbiological profiles of infectious keratitis: the Nottingham Infectious Keratitis Study. Eye.

[9] New definitions of S, I and R from 2019. https://www.eucast.org/newsiandr/. Accessed 30 Jan 2022

[10] Silvester A, Neal T, Czanner G (2016). Adult bacterial conjunctivitis: resistance patterns over 12 years in patients attending a large primary eye care centre in the UK. BMJ Open Ophthalmol.

[11] Hsiao CH, Sun CC, Yeh LK (2016). Shifting trends in bacterial keratitis in Taiwan: a 10-year review in a tertiary-care hospital. Cornea.

[12] Ferreira CS, Figueira L, Moreira-Gonçalves N (2018). Clinical and microbiological profile of bacterial microbial keratitis in a Portuguese tertiary referral center—where are we in 2015?. Eye Contact Lens.

[13] Orlans HO, Hornby SJ, Bowler ICJW (2011). In vitro antibiotic susceptibility patterns of bacterial keratitis isolates in Oxford, UK: a 10-year review. Eye.

[14] Soleimani M, Tabatabaei SA, Masoumi A (2021). Infectious keratitis: trends in microbiological and antibiotic sensitivity patterns. Eye.

[15] Xu S, Guo D, Liu X et al (2021) Ocular pathogens and antibiotic resistance in microbial keratitis over three years in Harbin, Northeast China. Acta Ophthalmol 1–7. 10.1111/aos.14789 10.1111/aos.14789 PMC9542897 33565253

[16] Steger B, Speicher L, Philipp W, Gasser T, Schmid E, Bechrakis N (2014). Effektivität antibiotischer Initialtherapie in der Behandlung der kontaktlinsenassoziierten bakteriellen Keratitis. Ophthalmologe.

[17] Asbell PA, Sanfilippo CM, Pillar CM et al (2015) Antibiotic resistance among ocular pathogens in the United States: five-year results from the Antibiotic Resistance Monitoring in Ocular Microorganisms (ARMOR) surveillance study. JAMA Ophthalmol 133. 10.1001/jamaophthalmol.2015.3888 10.1001/jamaophthalmol.2015.3888 26502312

[18] Oydanich M, Dingle TC, Hamula CL (2017). Retrospective report of antimicrobial susceptibility observed in bacterial pathogens isolated from ocular samples at Mount Sinai Hospital, 2010 to 2015. Antimicrob Resist Infect Control.

[19] Hsu HY, Ernst B, Schmidt EJ (2019). Laboratory results, epidemiologic features, and outcome analyses of microbial keratitis: a 15-year review from St. Louis Am J Ophthalmol.

[20] Bograd A, Seiler T, Droz S et al (2018) Bacterial and fungal keratitis : a retrospective analysis at a university hospital in Switzerland Bakterielle und mykotische Keratitis : eine retrospektive Analyse an einer Schweizer Universitätsklinik 10.1055/a-0774-7756 30616287

[21] Lichtinger A, Yeung SN, Kim P (2012). Shifting trends in bacterial keratitis in Toronto: an 11-year review. Ophthalmology.

[22] Konda N, Motukupally SR, Garg P (2014). Microbial analyses of contact lens-associated microbial keratitis. Optom Vis Sci.

[23] Böhm MRR, Prokosch V, Merté R-L (2011). Microbiological analysis in contact lens-associated keratits. Klin Monbl Augenheilkd.

[24] Andersson J, Vogt JK, Dalgaard MD (2021). Ocular surface microbiota in contact lens users and contact-lens-associated bacterial keratitis. Vis.

[25] Kowalski RP, Kowalski TA, Shanks RMQ (2013). In vitro comparison of combination and monotherapy for the empiric and optimal coverage of bacterial keratitis based on incidence of infection. Cornea.

[26] Petrillo F, Pignataro D, Di Lella FM (2021). Antimicrobial susceptibility patterns and resistance trends of staphylococcus aureus and coagulase-negative staphylococci strains isolated from ocular infections. Antibiotics.

[27] Asbell PA, Sanfilippo CM, Sahm DF, Decory HH (2020) Trends in antibiotic resistance among ocular microorganisms in the United States from 2009 to 2018. 38163:1–12. 10.1001/jamaophthalmol.2020.0155 10.1001/jamaophthalmol.2020.0155 PMC7146550 32271355

[28] Deguchi H, Kitazawa K, Kayukawa K (2018). The trend of resistance to antibiotics for ocular infection of *Staphylococcus aureus*, coagulase-negative staphylococci, and *Corynebacterium* compared with 10-years previous: a retrospective observational study. PLoS ONE.

[29] Peng MY, Cevallos V, McLeod SD et al (2018) Bacterial keratitis: isolated organisms and antibiotic resistance patterns in San Francisco. Cornea 37. 10.1097/ICO.0000000000001417 10.1097/ICO.0000000000001417 PMC5716884 29053557

[30] Chen Z (2008). Distribution of bacterial keratitis and emerging resistance to antibiotics in China from 2001 to 2004. Clin Ophthalmol.

[31] Afshari NA, Ma JJK, Duncan SM (2008). Trends in resistance to ciprofloxacin, cefazolin, and gentamicin in the treatment of bacterial keratitis. J Ocul Pharmacol Ther.

[32] Goldstein MH, Kowalski RP, Gordon YJ (1999). Emerging fluoroquinolone resistance in bacterial keratitis: a 5-year review. OPHTHA.

[33] Chang VS, Dhaliwal DK, Raju L, Kowalski RP (2015) Antibiotic resistance in the treatment of *Staphylococcus aureus* keratitis: a 20-year review. Cornea 34. 10.1097/ICO.0000000000000431 10.1097/ICO.0000000000000431 PMC4426026 25811722

[34] Deschênes J, Blondeau J (2015). Besifloxacin in the management of bacterial infections of the ocular surface. Can J Ophthalmol.

[35] Watson SL, Gatus BJ, Cabrera-Aguas M et al (2020) Bacterial Ocular Surveillance System (BOSS) Sydney, Australia 2017–2018. Commun Dis Intell 44. 10.33321/cdi.2020.44.86 10.33321/cdi.2020.44.86 33278871

[36] Laporte JR, Vidal X, Ballarín E, Ibáñez L (1998). Possible association between ocular chloramphenicol and aplastic anaemia — the absolute risk is very low. Br J Clin Pharmacol.

[37] Rosenthal RL, Blackman A (1965). Bone-marrow hypoplasia following use of chloramphenicol eye drops. JAMA.

[38] Ashfaq H, Maganti N, Ballouz D (2021). Procedures, visits, and procedure costs in the management of microbial keratitis. Cornea.

[39] Lee AE, Niruttan K, Rawson TM, Moore LSP (2019). Antibacterial resistance in ophthalmic infections: a multi-centre analysis across UK care settings. BMC Infect Dis.

